# Association between Single Nucleotide Polymorphisms of SULT1A1, SULT1C4, ABCC2 and Phase II Flavanone Metabolites Excretion after Orange Juice Intake

**DOI:** 10.3390/nu14183770

**Published:** 2022-09-13

**Authors:** Layanne Nascimento Fraga, Dragan Milenkovic, Franco Maria Lajolo, Neuza Mariko Aymoto Hassimotto

**Affiliations:** 1 Food Research Center (FoRC) and School of Pharmaceutical Sciences, University of São Paulo, São Paulo 05508-000, Brazil; 2 Department of Nutrition, University of California Davis, Davis, CA 95616-5270, USA

**Keywords:** sulfotransferase, ABC transporters, hesperidin, narirutin, flavanone metabolite, bioavailability, orange juice, polymorphism, SNP, interindividual variability

## Abstract

Citrus fruits and juices are a major source of dietary flavanones, and the regular consumption of these foods is inversely associated with the development of cardiometabolic diseases. However, the biological benefits depend on the bioavailability of these compounds, and previous studies have reported a large interindividual variability in the absorption and excretion of these compounds. Different factors, such as age, gender or genetic polymorphism of genes coding enzymes involved in the metabolism and transport of the flavanones, may explain this heterogeneity. This study aimed to assess the impact of single nucleotide polymorphism of sulfotransferases SULT1A1 and SULT1C4, and ABCC2 transporter genes on excretion of phase II flavanone metabolites in volunteers after 24 h of orange juice intake. Forty-six volunteers ingested a single dose of 500 mL of orange juice and 24-h urine was collected. The hesperetin and naringenin phase II metabolites were quantified in urine, and SNPs in SULT1A1, SULT1C4 and ABCC2 genes were genotyped. A significant (*p* < 0.05) relationship between the SNPs in these genes and the high excretion of phase II flavanone metabolites were observed. These results identified novel polymorphisms associated with higher absorption of flavanones, which may provide bases for future personalized nutritional guidelines for consuming flavanone-rich foods rich in these nutrients for better benefit from their health properties.

## 1. Introduction

Consumption of fruits and vegetables is inversely associated with development of diseases including cardiovascular, neurodegenerative and metabolic diseases [1,2]. These plant foods are consumed fresh, cooked or transformed into end products such as purees, compotes or juices. Among the most-consumed juice worldwide is orange juice [3]. Citrus drinks are rich sources of different micronutrients, and are a major source of dietary flavanones, a category of (poly)phenol compounds, mainly present as hesperidin in orange [4]. Epidemiological studies have shown that flavanone intake is associated with a lower incidence of cardiovascular disease (CVD) diseases and mortality [5,6]. These findings corroborate with results from preclinical studies which demonstrated that citrus flavanones can slow down development of atherosclerosis [7]. These atheroprotective effects have been related to the capacity of flavanones to modulate the expression of genes involved in the regulation of endothelial cell and vascular dysfunctions, particularly those regulating endothelial permeability [8,9]. Vascular health protective effects of citrus flavanones were furthermore reported in several randomized controlled clinical trials [10,11,12,13,14].

The ability of citrus flavanones to exert beneficial effects depends on their bioavailability. Citrus flavanones are not absorbed in the small intestine, and reach the colon where they are deglycosylated by the action of the resident gut microbiota α-rhamnosidase and β-glucosidase enzymes. Aglycons released can be absorbed, and are subject to phase II metabolism such as conjugation reactions including sulphatation and glucuronidation [15]. Flavanones were found circulating mainly as sulfated and glucuronidated metabolites and can be detected in urine. After consumption of orange juice, the main metabolites identified in the plasma and urine were hesperetin-7-glucuronide, hesperetin-3′-glucuronide, hesperitin-diglucuronide and hesperetin-3′-sulfate [10,16,17].

Different enzymes, encoded by genes in human genome, are involved in phase II metabolism of flavanones. Among the sulfotransferases enzymes, SULT1A1, SULT1C4, and to a smaller extent SULT1E1 and SULT1A3, demonstrated the highest catalytic efficiencies for the sulfonation of hesperetin [18,19]. SULT1A1 has been described to sulfonate other flavonoids as well [20,21,22]; nevertheless, SULT1A1 only catalyzes the formation of hesperetin 3′-*O*-sulfate, whereas SULT1C4 solely catalyzes the formation of hesperetin 7-*O*-sulfate [18]. The regioselectivity of flavonoid sulfonation appears to be dependent on the SULT isoenzyme as well as on the flavonoid studied: daidzein (4′,7-dihydroxyisoflavone) and genistein (4′,5,7-trihydroxyisoflavone) were described to be mainly sulfated by SULT1A1 at position 7 rather than at position 4′, while the hydroxyl moieties at both positions were sulfonated with similar effectiveness by SULT1E1 [21]. Incubations with the human cytosolic fractions demonstrated privileged sulfonation of position 3′ of hesperetin. Although SULT1A2, SULT1C4 and SULT1E1 present catalytic capacities based on expression levels, they are minor SULT isoforms in the intestine and liver. As a result, flavanones exist in the plasma mainly as sulfated and glucuronidated metabolites [23,24].

Furthermore, the multidrug resistance protein 2 (MRP2/ABCC2) is part of the ABC transporters involved in the efflux of flavanone metabolites back to the intestinal lumen, which may limit the bioavailability of these compounds [25,26]. It has been reported that hesperetin 7-glucuronide and hesperetin 3′-glucuronide interact with human ABCC2 [26].

The mean peak plasma concentrations of flavanones vary between 0.1 and 1 μM for intakes ranging from a 150 g orange to 500 mL of orange juice. Urinary excretion of flavanones mainly occurs during the 24 h following ingestion, peaking between 6 and 12 h. The rate of urinary excretion, expressed as a percentage of the total intake, indicates that flavanones are among the most bioavailable dietary polyphenols [16]. Thus, after consumption of an orange (as juice or whole fruit), the relative urinary excretion of hesperetin varies between 1.7% and 6.4% [27].

However, in recent years, high interindividual variability in metabolism of polyphenols, including citrus flavanones, have been reported. It has been observed that it would be possible to stratify volunteers into high, medium and low phase II conjugates excretors [10,28,29]. Different factors, such as age, diet, lifestyle, health status, medication and potentially genetic background, may present the main determinants of variation in absorption, distribution, metabolism and excretion (ADME) observed across individuals [30]. However, the role of these factors is not yet well demonstrated. This variability in metabolism is one of the factors of inter-individual variability in biological response to polyphenols regarding cardiometabolic health outcomes [31]. Therefore, better understanding as to why some polyphenol compounds are more bioavailable and consequently can exert health properties in some individuals but not, or less, in others is vital for a consideration of these bioactives in future approaches of personalized nutrition [32].

Thus, the aim of this study was to assess the impact of single nucleotide polymorphisms in genes coding for phase II enzyme SULT1A1 and SULT1C4 and transporter ABCC2 on excretion of flavanones phase II metabolites in volunteers after 24 h of orange juice intake.

## 2. Materials and Methods

### 2.1. Orange Juice

Pasteurized orange juice (*Citrus sinensis* L. Osbeck var. Pera), obtained in June of 2019, was supplied by Fundecitrus (Araraquara, Brazil), located in southeastern São Paulo state. The characterization of orange juice was performed in our previous study [10]. The juice (9.56°Brix) was poured into 1 L flasks and immediately stored at −20 °C. Total soluble sugar content, including sucrose, glucose and fructose, was 5.03 g per 100 mL, representing 25 kcal per 100 mL. The orange juice contained 25.46 mg per 100 mL of hesperidin and 1.95 mg per 100 mL of narirutin.

### 2.2. Subjects

A total of 110 subjects were enrolled in the study according to the inclusion and exclusion criteria, and 85 completed the study. Among them, 74 delivered 24-h urine and 46 subjects were included in this study. The inclusion criteria were healthy volunteers classified according to body mass index (BMI) as either eutrophic (BMI = 18.50–24.99 kg m^−2^) and overweight (BMI = 25.00–29.9 kg m^−2^), both sexes, aged from 19 to 40 years. Exclusion criteria were cardiovascular, gastrointestinal, hepatic or renal diseases; diabetes; alcohol consumption; vegetarianism; use of vitamin and mineral supplements, or supplements containing plant foods or extracts, antibiotics, antacids, medications for diarrhea or constipation; use of medications for blood pressure or lipid control; smoking; aversion to orange juice; pregnancy, breastfeeding mothers or those taking hormone therapy for menopause and high-performance athletes. The study was carried out in accordance with the Declaration of Helsinki, and all procedures were approved by the Ethical Committee of the School of Pharmaceutical Science-University of São Paulo, São Paulo (CAAE 14344819.0.0000.0067) and the Ethical Committee of the University Hospital of São Paulo (CAAE 14344819.0.3001.0076). Written informed consent was obtained before the commencement of the study. Clinical trial settings and data collection were performed at the clinical unit of the University Hospital of São Paulo, Brazil.

### 2.3. Study Design

The primary aim of the present study was to assess the relationship between inter-individual variability in the excretion of citrus flavanone and the effect on cardiometabolic biomarkers, for which the results have been published elsewhere [10]. The second outcome was to examine the association between single nucleotide polymorphisms of phase II enzyme, SULT and transporter ABCC2 and flavanone metabolites excretion. Three days before the intervention, volunteers were instructed not to consume citrus-containing foods (orange, lemon, grapefruit, citrus juice) and to follow a low-polyphenol diet avoiding strawberries, passion fruit, coffee, chocolate, wine and teas. On the first day of the intervention, overnight fasted (12 h) volunteers had blood, feces and urine collected, blood pressure (BP) measured and anthropometric parameters assessed, and a 24 h dietary recall was performed. Furthermore, volunteers ingested 500 mL of orange juice in a single dose, and urine was collected over 24 h at intervals of 0–4 h, 4–8 h, 8–12 h and 12–24 h in a collecting bottle containing 0.5 g of ascorbic acid. Urine samples were stored at −80 °C until analysis.

### 2.4. Identification and Quantification of Urine Phase II Conjugates and Phenolic Acids

Citrus phase II flavanone metabolites were previously identified and quantified in Fraga et al. [10], as described by Nishioka et al. [29]. Briefly, urine samples were centrifuged at 14,000× *g* for 5 min at 4 °C and filtered using a 0.22 μm PVDF filter (Millipore Ltd., Bedford, MA, USA). Urine samples were analyzed by HPLC (Prominence, Shimadzu, Japan) coupled to the mass spectrometer type qTOF, model Compact (Bruker Daltonics, Germany), and by UPLC-Nexera LC-30AD (Shimadzu, Kyoto, Japan) coupled to an EVOQ™ triple quadrupole mass spectrometer (Bruker Daltonics, Germany). Separation of each compound was performed on a Poroshell 120 C18 column (2.7 μm particle, 100 × 3.0 mm) (Agilent, CA, USA), equipped with a 20 mm × 4.0 mm guard column. The eluates were monitored at 280 nm and 525 nm by a diode array detector (DAD) and, subsequently, an electrospray ionization-mass spectrometry (ESI-MS). The mass spectrometer was operated in negative mode, source voltage 3500 V, cone temperature 350 °C, cone gas flow 20 L/min, heated probe temperature 350 °C, probe gas flow 40 units, nebulizergas flow 50 units. Phase II conjugates and phenolic acids were identified by the similarity of the mass spectra profile compared to the literature data. Naringenin 7-glucuronide, hesperetin 7-glucuronide and hesperetin 3′-glucuronide, kindly donated by Dr Paul Kroon and Dr Paul Needs (Quadram Institute, UK), were used to confirm the identity of some metabolites. For quantification, calibration curves of the available standards were performed in concentrations ranging from 0.002 to 20μg mL^−1^ (R2 = 0.999). Detection limit was 0.002 μg/mL and quantification limit was 0.008 μg/mL. All hesperetin and naringenin phase II metabolites were quantified using the calibration curve of hesperetin 7-glucuronide and naringenin 7-glucuronide, respectively.

### 2.5. DNA Isolation

The genomic DNA was isolated from their peripheral blood collected in tubes containing ethylene- diamine tetra acetic acid (EDTA) anticoagulants. DNA was extracted using PureLink™ Genomic DNA Mini Kit (Thermo Fisher Scientific, Waltham, MA, USA) following manufacture’s recommendations. The concentration and the purity of the DNA samples were evaluated with a Nano Drop device (Thermo Fisher Scientific, Waltham, MA, USA), the 260/280 nm absorbance ratio between 1.7–2 was considered acceptable, and a 1% agarose gel was made for all the samples.

### 2.6. Determination of SNP Genotypes

The DNA samples extracted were analyzed for identification of single-nucleotide polymorphisms (SNPs) in genes related to absorption, metabolism, distribution and excretion of phase II flavanone metabolites. The analysis was performed at the LGC, Biosearch Technologies laboratory in Hoddesdon. The laboratory uses its proprietary KASP^®^ genotyping technology. KASP™ genotyping assays are based on competitive allele-specific PCR and enable bi-allelic scoring of single nucleotide polymorphisms (SNPs) and insertions and deletions (Indels) at specific loci as informed by the laboratory (https://segolip.ilri.org/kasp_genotyping (accessed on 1 June 2022)). The SNPs and primer used are described in Table 1. The KASP Assay mix contains three assay-specific non-labelled oligos: two allele-specific forward primers and one common reverse primer. The allele-specific primers each harbor a unique tail sequence that corresponds with a universal FRET (fluorescence resonant energy transfer) cassette. If the genotype at a given SNP is homozygous, only one of the two possible fluorescent signals will be generated. If the genotype is heterozygous, a mixed fluorescent signal will be generated.

### 2.7. Statistical Analyses

The Hardy–Weinberg equilibrium (HWE) was applied to assess the deviation of the genotype or allele frequency. Multiple logistic regression models (codominant, dominant, recessive, over dominant and log-additive) were applied to analyze the correlation between the SNP data and disease phenotype (odds ratio) with 95% confidence interval was calculated using the SNPStats software (https://www.snpstats.net/start.htm (accessed on 12 June 2022) (doi.org/10.1093/bioinformatics/btl268). *p* values less than 0.05 were considered significant.

## 3. Results

### 3.1. Study Population Characteristics

To investigate the association of SNPs in SULT1A1, SULT1C4 and ABCC2 with total phase II flavanone metabolites in the studied population, a subset of 46 subjects were enrolled in this study according to the previous stratification on phase II flavanone metabolites excretors (Fraga et al., 2021), including 25 high-excretors and 21 low-excretors. The general characteristics were shown in Table 2.

### 3.2. Association of ABCC2_rs8187710 with Phase II Flavanone Metabolites Excretion

**Figure 1:**
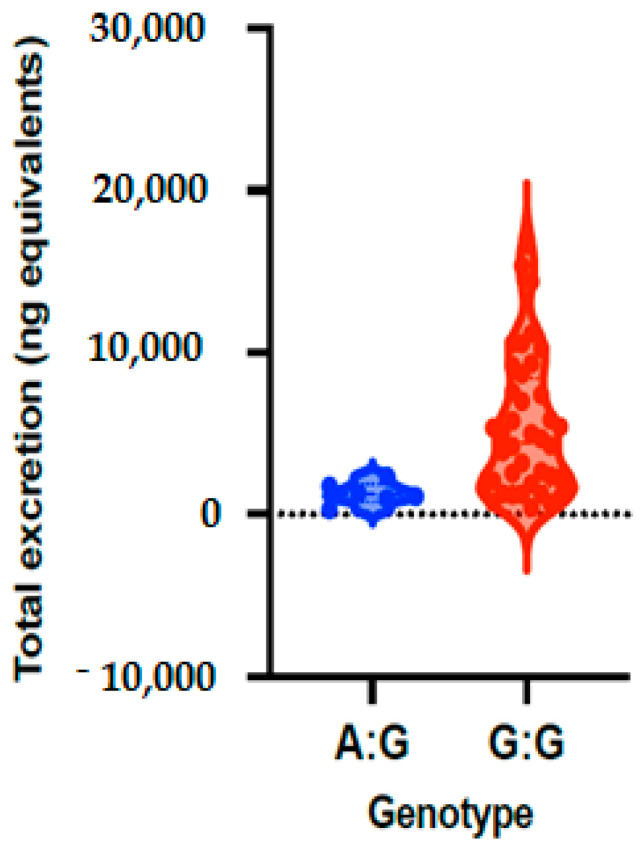
Total phase II flavanone metabolite concentrations in urine depending on genotypes of ABCC2_rs8187710 polymorphism. Bars in the box show the first quartile, the third quartile and median and whisker charts represent the minimum to maximum values.

The allele and genotype frequencies of ABCC2_rs8187710 are presented in Table 3 and Figure 1. The distribution of genotypes was consistent with HWE in both groups (*p* > 0.05) (Supplementary Table S1). The frequency of allele G is 93% and allele A is 7% in the studies volunteers, with allele frequency of 88% for G and 12% for A in low excretors and 98% for G and 2% for A in high excretors. The frequency of the genotypes of ABCC2 gene polymorphism (rs8187710) for GA is 13% and for GG is 87% in all volunteers, a frequency that was observed to be 24% for GA and 76% for GG in volunteers with low excretors, and 4% for GA and 96% for GG in high excretors. Genetic models were applied to analyze the associations between ABCC2 polymorphism and excretion of phase II flavanone metabolites in urine of volunteers after 24 h of orange juice intake. The results of the genetic models showed that ABCC2 polymorphism (rs8187710) is significantly associated with quantity of phase II flavanone metabolites excreted in urine, with OR = 0.13; 95% CI = 0.01–1.25, *p* = 0.041 (Table 4).

### 3.3. Association of SULT1A1_rs3760091 with Phase II Flavanone Metabolites Excretion

The allele and genotype frequencies of SULT1A1_rs3760091 are presented in Table 5. The distribution of genotypes was consistent with HWE in both groups (*p* > 0.05) (Supplementary Table S1). We observed that allele frequency of C is 61% and that of G is 39% in all studied volunteers. The allele frequency of C was observed to be 55% in low excretor volunteers and 66% in high excretors, with frequency of G being 45% in low excretors and 34% in high excretors. Regarding the genotype frequency, we observed that frequencies of CC, CG and GG were 61%, 39% and 11%, respectively, in the total studied population. The frequency was 55%, 45% and 19% for CC, CG and GG, respectively, in low excretors and 66%, 34% and 4%, respectively, in high excretors (Table 5).

**Figure 2:**
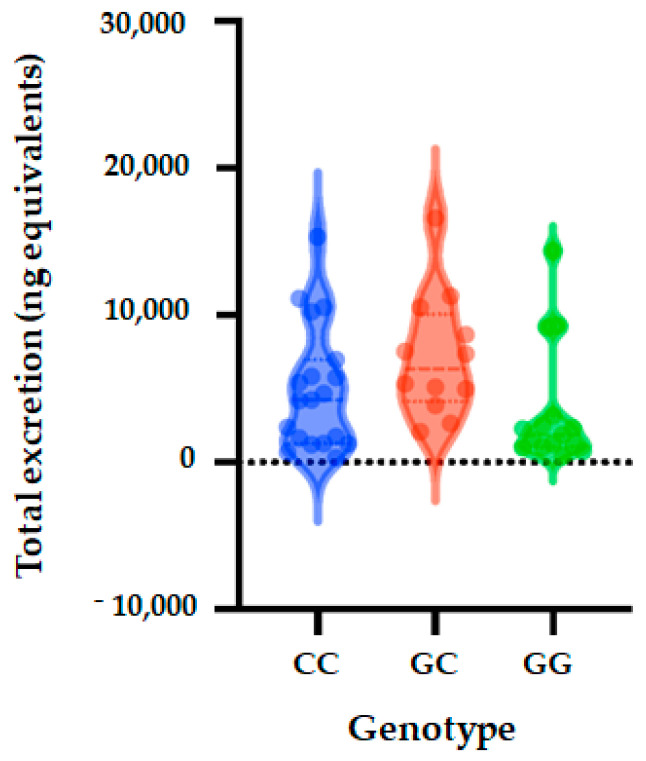
Total metabolite concentrations in urine depending on genotypes of SULT1A1_rs3760091 polymorphism. Bars in the box show the first quartile, the third quartile, and median and whisker charts represent the minimum to maximum values.

Five genetic models (codominant, dominant, recessive, over dominant and log-additive) were applied to analyze the associations between SULT1A1 polymorphism and excretion of phase II flavanone metabolites in the urine of volunteers after 24 h of orange juice intake. The genetic models analysis showed that SULT1A1 polymorphism (rs3760091) is not significantly associated with quantity of phase II flavanone metabolites excreted in urine (codominant (OR = 0.91; 95% CI = 0.25–3.31, *p* = 0.25), dominant (OR = 0.71; 95% CI = 0.20–2.48, *p* = 0.59), over dominant (OR = 0.18; 95% CI = 0.02–1.73, *p* = 0.095), and log-additive (OR = 0.55; 95% CI = 0.21–1.45, *p* = 0.22) (Table 6 and Figure 2).

### 3.4. Association of SULT1A1_rs4788068 with Phase II Flavanone Metabolites Excretion

Following genotyping, we observed that the distribution of genotypes was consistent with HWE in both groups (*p* > 0.05) (Supplementary Table S1). For this SNP, the allele frequency of C was observed to be 66% and that of T to be 34% in all of the studied population. The allele frequency of C was observed to be 50% in low excretors and 80% in high excretors. The allele frequency was 50% in low excretors for allele T and of 20% in high excretors. We observed that in the studied population, the CC, CT and TT genotype frequencies were 43%, 46% and 11% in total population. The genotype frequencies were 19%, 62% and 19% for CC, CT and TT, respectively, in low excretor and 64%, 32% and 4% in high secretors (Table 7).

**Figure 3:**
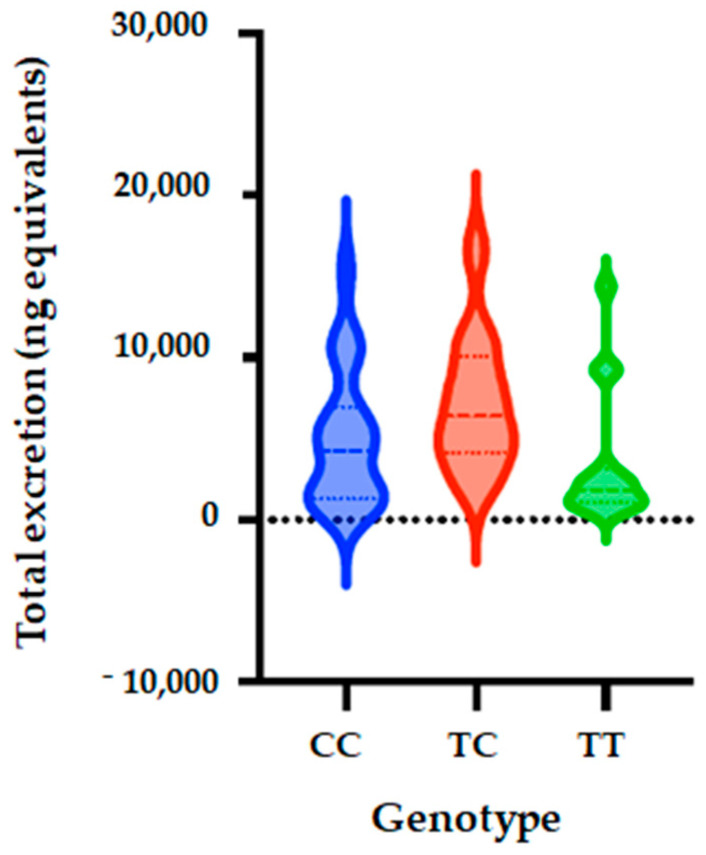
Total metabolite concentrations in urine depending on genotypes of SULT1A1_rs4788068 (B) polymorphism. Bars in the box show the first quartile, the third quartile, and median and whisker charts represent the minimum to maximum values.

Five genetic models (codominant, dominant, recessive, over dominant and log-additive) were also applied to analyze the associations between SULT1A1_rs4788068 polymorphism and excretion of phase II flavanone metabolites in the urine of volunteers after 24 h of orange juice intake. The genetic model analysis showed that SULT1A1 polymorphism (rs4788068) is significantly associated with quantity of phase II flavanone metabolites excreted in urine. The analyses showed that for the codominant model, OR is 0.15; 95% CI = 0.04–0.63, *p* = 0.0053); dominant model: OR = 0.18; 95% CI = 0.02–1.73, *p* = 0.0017); over dominant model showed OR of 0.29; 95% CI = 0.09–0.98, *p* = 0.041), and log-additive (OR = 0.2; 95% CI = 0.07–0.61, *p* = 0.0015) (Table 8, Figure 3).

### 3.5. Association of SULT1C4_rs1402467 with Phase II Flavanone Metabolites Excretion

**Figure 4:**
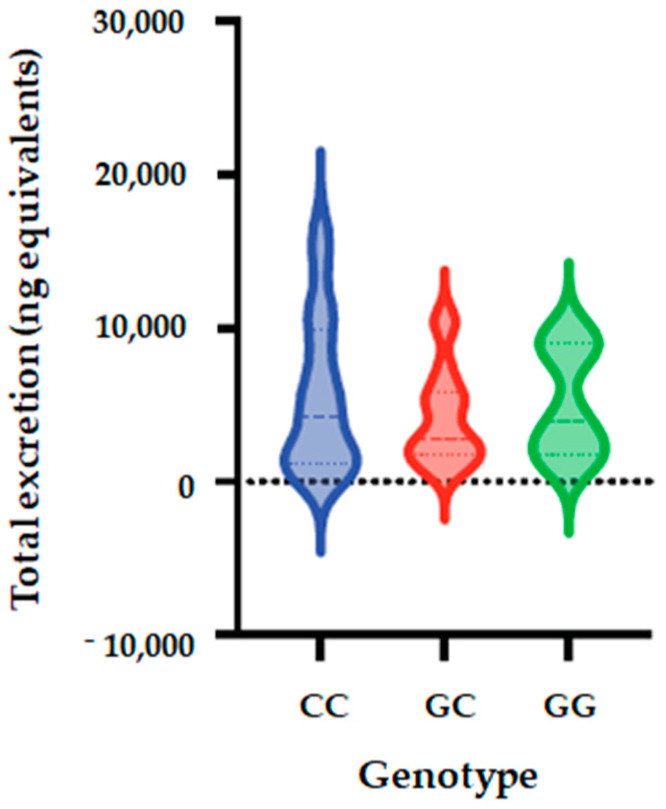
Total metabolite concentrations in urine depending on genotypes of SULT1C4_rs1402467 polymorphism. Bars in the box show the first quartile, the third quartile, and median and whisker charts represent the minimum to maximum values.

The allele and genotype frequencies of SULT1C4_ rs1402467 are presented in Table 9. The distribution of genotypes that is presented is inconsistent with HWE in both groups (*p* < 0.05) (Supplementary Table S1), with one volunteer who cannot be genotyped. We observed that the frequency of C allele was 70% in the total population, 52% in low excretors and 85% in high excretors. Regarding allele G, its frequency was 30% in all populations, 48% in low excretors and 15% in high excretors. We observed that genotype CC frequency was 56% in total population, 29% in low excretor and 79% for high excretors; the frequency of CG was 29%, 48% and 12%. For the total population, low excretors and high excretors, respectively, while the frequency of GG was 29%, 48% and 12%, respectively. Using five genetic models (codominant, dominant, recessive, over dominant and log-additive), we observed a significantly association between SULT1C4_ rs1402467 polymorphism and excretion of phase II flavanone metabolites in urine of volunteers after 24 h of orange juice intake. The analyses showed that for the codominant model, OR is 0.09; 95% CI = 0.02–0.46, *p* = 0.0022); dominant model: OR = 0.11; 95% CI = 0.03–0.41, *p* = 5 × 10^−4^); over dominant model showed OR of 0.16; 95% CI = 0.04–0.69, *p* = 0.0084), and log-additive (OR = 0.25; 95% CI = 0.09–0.68, *p* = 0.0021) (Table 10, Figure 4).

### 3.6. Haplotype Analysis

Together with identification of associations between individual SNPs and phase II flavanone metabolites excretion, we also assessed the frequency of haplotypes of these 4 SNPs and the associations with the phenotype. Estimated haplotype frequencies are presented in the Table 11. The estimated cumulated frequencies vary from 40% for GCCC for rs8187710/rs3760091/rs4788068/rs1402467 SNPs, 60% for GGCC, 74.3% for GCTG to over 98% for GCCG. The association analyses between haplotypes and phenotype revealed 2 haplotypes significantly associated with high excretion of phase II flavanone metabolites following 24 h of intake of orange juice. We observed that GGCC haplotype for ABCC2_rs8187710/SULT1A1_rs3760091/SULT1A1_rs4788068/SULT1C4_rs1402467 is significantly associated with phenotype high excretors with OR = 0.07, 95% CI = 0.01–0.81, *p* value = 0.039. For haplotype GCTG for ABCC2_rs8187710/SULT1A1_rs3760091/SULT1A1_rs4788068/SULT1C4_rs1402467, a significant association was also observed with OR = 0.01, 95% CI = 0.00–0.17, *p* value = 0.0038. Global haplotype association *p* value observed is 0.00044 (Table 12).

## 4. Discussion

Hesperidin and narirutin are the major flavanones found in citrus, and previous studies reported that regular consumption of these foods is related to a reduction in the occurrence of chronic non-communicable diseases such as obesity, diabetes and cardiovascular diseases [5,6]. These compounds are absorbed when they reach the colon after being hydrolyzed by the resident microbiota, and the released hesperetin and naringenin aglycones are absorbed. The aglycones can be conjugated to the glucuronic acid and/or to sulfate through the action of phase II metabolism enzymes SULT and UGT in the colonocyte, then transported to the liver where they can undergo first conjugation or second conjugation by SULT and UGT [15,33,34]. These metabolites then reach target tissues and are excreted in the urine, mainly as flavanones conjugated to sulfate and glucuronic acid [16,17]. In addition to the transport of these compounds between membranes, efflux back into the intestinal lumen, as well as their excretion, is mediated by specific transporters, the ABC transporters [15].

Nonetheless, a large interindividual in the bioavailability of flavanones has been reported. Previously, stratifications were proposed according to the total number of metabolites excreted in the urine of volunteers after ingestion of orange juice, to high, medium and low excretors [10,28,29]. Several factors have been reported as probable determinants of heterogeneity observed in the excretion of these compounds, such as sex, age, intestinal microbiota, BMI and polymorphisms in the genes of key enzymes in the metabolism and transport of these [28,29]. Brett et al. [35] observed a weak but significant correlation between the age of participants and flavanone metabolism. In contrast, in our previous study, none of these factors were determinants of the variability found in the total excretion of flavanone metabolites, even though high heterogeneity in the excretion was observed [10].

In this way, we select the MRP2/ABCC2 (rs8187710), SULT1A1 (rs3760091 and rs4788068) and SULT1C4 (rs1402467) genes and respective SNPs, based on previous published studies which have reported that there is a large inter-individual variability in the excretion of flavonoids potentially influenced by the SNP in the genes of ABC transporters and/or in the genes of phase II metabolism enzymes [10,29,30]. Previously, flavanone hesperetin in vitro and in vivo were shown to interact with transporters such as MRP2/ABCC2 and be highly sulfated by SULT1A1, SULT1C4, and to a smaller extent SULT1E1 and SULT1A3 [18,26]. Moreover, previous studies have shown that the presence of SNPs in the ABCC2 gene as well as in the SULT affects the bioavailability of epicatechins [36], yet a brief release from the COB study, with chocolate, orange and blackberry, identified these SNPs in female volunteers who consumed these foods, but to date, no direct relationship between the presence of these identified SNPs and the bioavailability of the compounds present in these foods has been reported [37].

In addition, no study has shown if the presence of SNPs in these genes may affect the excretion of flavanones after orange juice ingestion in humans, which could in part explain the large interindividual variability found in the excretion of these compounds.

Previously, very few previous studies have investigated other factors, such as SNPs in genes of key enzymes the ADME of polyphenols to identify determinants of the variability found in the excretion of these compounds. It was reported in an in vitro study that the SULT1A1*2 variant is less efficient than SULT1A1*1 in conjugating resveratrol, apigenin and epicatechin [22]. It was also observed that polymorphism in the UGT1A6 gene directly impacted the conjugation of resveratrol to glucuronic acid, and a positive correlation was observed between the SNP in UGT1A6 and glucuronidation kinetics of cis-resveratrol [38]. Another study in humans reported that CYP1A2*1F affects the metabolism of caffeine and that the individuals with the CYP1A2*1F allele presented lower metabolism of caffeine concerning the wild-type variant [39] (In addition, SNPs in genes of phase II enzyme and transporters was observed to affect the ADME of green tea polyphenols. In volunteers carrying the wild type of ABCC2 and OATP1B1, reduction of relative bioavailability of epigallocatechin gallate compared to carriers of the variant allele was observed and volunteers with UGT1A1*28 variant presented reduction of relative bioavailability and clearance of epigallocatechin [36].

Few studies have been reported regarding the SNPs used in our study. One study showed that interindividual variability in onset of menopause and symptoms before initiation of hormone therapy can be explained in part by genetic variation in SULT1A1 and may represent a step toward individualizing hormonal treatment decisions [40]. Regarding the SULT1A1_rs4788068, it has been described that this SNP is significantly associated with the gene expression, as well as enzymatic activity of SULT1A1, suggesting genotype-specific manner [41]. Several studies have suggested association between ABCC2_rs8187710 and potential cardiotoxicity of certain chemotherapeutic agents. A systematic review and meta-analysis of 28 studies examining the association of genetic variants and anthracycline-induced cardiotoxicity discovered significant increase in anthracycline-induced cardiotoxicity and ABCC2_rs8187710 polymorphism [42]. ABCC2_rs8187710 polymorphism was associated with a higher accumulation of lopinavir, a protease inhibitor used in the treatment of HIV-infected patients, in peripheral blood mononuclear cells of HIV-treated patients, genetic polymorphism could explain a large part of the interindividual variability in pharmacokinetics of this drug [43]. The ABCC2_rs717620 polymorphism was also found to be associated with an increased risk of hyperbilirubinemia, drug-induced liver injury [44]. Therefore, polymorphism in this gene is associated with significant impact on uptake of xenobiotics and can affect the pharmacokinetics (ADME, and toxicity) of various (anticancer) drugs [45]. These finding can therefore be corroborated with our finding that we observed a significant association between the GGCC and GCTG haplotypes for ABCC2_rs8187710/SULT1A1_rs3760091/SULT1A1_rs4788068/SULT1C4_rs1402467 with phenotype high excretors (Table 11), suggesting that polymorphism of these genes can significantly impact absorption and excretion of orange flavanones and differentiate high and low excretors of these metabolites.

**Figure 5:**
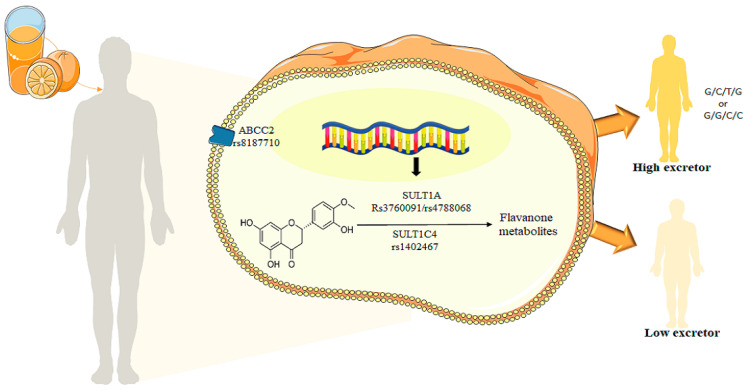
Total metabolite concentrations in urine depending the GGCC and GCTG haplotypes for ABCC2_rs8187710/SULT1A1_rs3760091/SULT1A1_rs4788068/SULT1C4_rs1402467 with phenotype high excretors.

Results from abundant population-based and clinical trials provided a consensus on the beneficial effects of diets rich in plant-based foods for the prevention of the recommendations of plant foods that are promoted at a population level with a “one-size fits-all” approach. Studies have, however, revealed inter-individual variability in the effects of plant foods [31] and consequently does not ensure that everyone benefits from the protective nutrients provided by these foods. Among the nutrients present in fruits and vegetables are polyphenols, which play important roles in the health effects of plant foods [11,12]. The bioavailability of these bioactives, including absorption and excretion, also present inter-individual variability, and several potential factors have been identified, such as genetic background, sex and age [46]. This study is one of the first studies identifying genetic polymorphism potentially involved in the inter-individual variability in absorption and secretion of phase II flavanone metabolites following intake of orange juice (Figure 5).

## 5. Conclusions

The obtained findings provide novel data for developing future personalized nutrition. Personalized nutrition is an approach that uses individual characteristics, such as lifestyle, anthropometry, phenotypes or genetic background, for specific dietary advice for better benefits of foods and prevention of diseases [47]. The results from this study could be used to identify individuals possessing genetic polymorphism associated with higher absorption of flavanones and provide them with personalized guidelines to consume foods rich in these nutrients and benefit from their health properties.

## Figures and Tables

**Table 1:** Single Nucleotide Polymorphisms and primers used.

Genes	SNPID	Allele Y	Allele X	Sequence
SULT1C4	rs1402467	G	C	AATGGCCTTACACGA[C/G]CATGGAGGATTTTA
SULT1A1	rs3760091	G	C	GACTCAGCAAAAGCA[C/G]CAGGCCTAGGCAGG
SULT1A1	rs4788068	T	C	TCACTTGTCAAGTAG[C/T]CTGGGACTACAGGT
ABCC2	rs8187710	A	G	GGAAGATTATAGAGT[G/A]GCGGCAGCCCTGAA

**Table 2:** Clinical and anthropometric characteristics of the population.

	Total	Low Excretor	High Excretor
Number of volunteers	46	21	25
Age (mean) years	26.26 ± 4.50	25.57 ± 4.24	26.64 ± 4.72
Age (range) years	19–38	19–35	19–38
Men	20	7	13
Women	26	14	12
Weight (Kg)	66.09 ± 11.08	65.80 ± 8.68	66.34± 12.92
BMI (Kg/m^2^)	23.23 ± 2.54	23.12± 2.69	23.32 ± 2.46
Triglycerides (mg/dL)	90.82 ± 45.19	97.85 ± 42.20	84.92 ± 47.59
Total cholesterol (mg/dL)	168.56 ± 29.45	164.61 ± 32.72	171.88 ± 26.61
LDL (mg/dL)	88.06 ± 27.86	81.61 ± 27.03	93.48 ± 27.93
HDL (mg/dL)	62.32 ± 17.02	63.38 ± 13.64	61.44 ± 19.66
SBP (mmHg)	115.54 ± 13.04	115.19 ± 13.54	115.84 ± 12.88
DBP (mm Hg)	68.05 ± 8.41	66.52 ± 7.98	69.36 ± 9.33

**Table 3:** Genotypes and allele frequencies of ABCC2_rs8187710 polymorphism in studied population.

ABCC2_rs8187710							
		All Subjects		Low Excretor		High Excretor	
	Allele	Count	Proportion	Count	Proportion	Count	Proportion
*Allele frequencies*	G	86	0.93	37	0.88	49	0.98
	A	6	0.07	5	0.12	1	0.02
*Genotype frequencies*	G/A	6	0.13	5	0.24	1	0.04
	G/G	40	0.87	16	0.76	24	0.96

**Table 4:** Analysis of association of ABCC2_rs8187710 polymorphism with the excretion of phase II flavanone metabolites in urine of studied volunteers after single dose orange juice.

ABCC2_rs8187710 Association with Metabolism							
Model	Genotype	Low Excretor	High Excretor	OR (95% CI)	*p*-Value	AIC	BIC
	G/G	16 (76.2%)	24 (96%)	1	0.041	63.2	66.9
	A/G	5 (23.8%)	1 (4%)	0.13 (0.01–1.25)			

Abbreviations definitions **AIC**: Akaike; **BIC**: Bayesian; **OR**: odds ratio; **95%CI: 95%** Confidence Interval.

**Table 5:** Genotypes and allele frequencies of SULT1A1_rs3760091 polymorphism in studied population.

SULT1A1_rs3760091							
		All Subjects		Low Excretor		High Excretor	
	Allele	Count	Proportion	Count	Proportion	Count	Proportion
*Allele frequencies*	C	56	0.61	23	0.55	33	0.66
	G	36	0.39	19	0.45	17	0.34
*Genotype frequencies*	C/C	56	0.61	23	0.55	33	0.66
	C/G	36	0.39	19	0.45	17	0.34
	G/G	5	0.11	4	0.19	1	0.04

**Table 6:** Analysis of association of SULT1A1_rs3760091 polymorphism with the excretion of phase II flavanone metabolites in urine of studied volunteers after a single dose of orange juice.

SULT1A1_rs3760091 Association with Metabolism							
Model	Genotype	Low Excretor	High Excretor	OR (95% CI)	*p*-Value	AIC	BIC
Codominant	C/C	6 (28.6%)	9 (36%)	1	0.25	66.6	72.1
	G/C	11 (52.4%)	15 (60%)	0.91 (0.25–3.31)			
	G/G	4 (19.1%)	1 (4%)	0.17 (0.01–1.88)			
Dominant	C/C	6 (28.6%)	9 (36%)	1	0.59	67.1	70.8
	G/C-G/G	15 (71.4%)	16 (64%)	0.71 (0.20–2.48)			
Recessive	C/C-G/C	17 (81%)	24 (96%)	1	0.095	64.6	68.3
	G/G	4 (19.1%)	1 (4%)	0.18 (0.02–1.73)			
Overdominant	C/C-G/G	10 (47.6%)	10 (40%)	1	0.6	67.2	70.8
	G/C	11 (52.4%)	15 (60%)	1.36 (0.42–4.40)			

**Table 7:** Genotypes and allele frequencies of SULT1A1_rs4788068 polymorphism in studied population.

SULT1A1_rs4788068							
		All Subjects		Low Excretor		High Excretor	
	Allele	Count	Proportion	Count	Proportion	Count	Proportion
*Allele frequencies*	C	61	0.66	21	0.5	40	0.8
	T	31	0.34	21	0.5	10	0.2
*Genotype frequencies*	C/C	20	0.43	4	0.19	16	0.64
	C/T	21	0.46	13	0.62	8	0.32
	T/T	5	0.11	4	0.19	1	0.04

**Table 8:** Analysis of association of SULT1A1_rs4788068 polymorphism with the excretion of phase II flavanone metabolites in urine of studied volunteers after single dose of orange juice.

SULT1A1_rs4788068 Association with Metabolism							
Model	Genotype	Low Excretor	High Excretor	OR (95% CI)	*p*-Value	AIC	BIC
Codominant	C/C	4 (19.1%)	16 (64%)	1	**0.0053**	58.9	64.4
	T/C	13 (61.9%)	8 (32%)	**0.15 (0.04–0.63)**			
	T/T	4 (19.1%)	1 (4%)	**0.06 (0.01–0.72)**			
Dominant	C/C	4 (19.1%)	16 (64%)	1	**0.0017**	57.6	61.2
	T/C-T/T	17 (81%)	9 (36%)	**0.13 (0.03–0.52)**			
Recessive	C/C-T/C	17 (81%)	24 (96%)	1	**0.095**	64.6	68.3
	T/T	4 (19.1%)	1 (4%)	**0.18 (0.02–1.73)**			
Overdominant	C/C-T/T	8 (38.1%)	17 (68%)	1	**0.041**	63.3	66.9
	T/C	13 (61.9%)	8 (32%)	**0.29 (0.09–0.98)**			

In bold the significant difference *p* < 0.05.

**Table 9:** Genotypes and allele frequencies of SULT1A1_rs1402467 polymorphism in studied population.

SULT1C4_rs1402467							
		All Subjects		Low Excretor		High Excretor	
	Allele	Count	Proportion	Count	Proportion	Count	Proportion
*Allele frequencies*	C	63	0.7	22	0.52	41	0.85
	G	27	0.3	20	0.48	7	0.15
*Genotype frequencies*	C/C	25	0.56	6	0.29	19	0.79
	C/G	13	0.29	10	0.48	3	0.12
	G/G	7	0.16	5	0.24	2	0.08

**Table 10:** Analysis of association of SULT1A1_ rs1402467 polymorphism with the excretion of phase II flavanone metabolites in urine of studied volunteers after single dose of orange juice.

SULT1C4_rs1402467 Association with Metabolism							
Model	Genotype	Low Excretor	High Excretor	OR (95% CI)	*p*-Value	AIC	BIC
Codominant	C/C	6 (28.6%)	19 (79.2%)	1			
	G/C	10 (47.6%)	3 (12.5%)	**0.09 (0.02–0.46)**	**0.0022**	56	61.4
	G/G	5 (23.8%)	2 (8.3%)	**0.13 (0.02–0.83)**			
Dominant	C/C	6 (28.6%)	19 (79.2%)	1	**5.00 × 10^−4^**	54	57.7
	G/C-G/G	15 (71.4%)	5 (20.8%)	**0.11 (0.03–0.41)**			
Recessive	C/C-G/C	16 (76.2%)	22 (91.7%)	1	0.15	64.1	67.7
	G/G	5 (23.8%)	2 (8.3%)	0.29 (0.05–1.69)			
Overdominant	C/C-G/G	11 (52.4%)	21 (87.5%)	1	**0.0084**	59.2	62.8
	G/C	10 (47.6%)	3 (12.5%)	**0.16 (0.04–0.69)**			

In bold the significant difference *p* < 0.05.

**Table 11:** Haplotype frequencies for SNPs for ABCC2_rs8187710/SULT1A1_rs3760091/SULT1A1_rs4788068/SULT1C4_rs1402467.

Haplotype Frequencies Estimation								
	rs8187710	rs3760091	rs4788068	rs1402467	Total	Group.0	Group.1	Cumulative Frequency
1	G	C	C	C	0.4015	0.1617	0.5586	0.4015
2	G	G	C	C	0.2022	0.2861	0.1983	0.6037
3	G	C	T	G	0.1393	0.2664	0.0468	0.743
4	G	G	T	G	0.1034	0.1172	0.0717	0.8464
5	G	G	T	C	0.0479	0.0244	0.0499	0.8943
6	A	G	C	C	0.0348	0	0.02	0.9291
7	A	C	T	G	0.0275	0.0673	NA	0.9566
8	G	C	C	G	0.0245	0.025	0.023	0.9811
9	G	C	T	C	0.0159	NA	0.0315	0.9971
10	A	G	T	C	0.0029	0.0244	NA	1
11	A	G	T	G	0	2.00 × 10^−4^	NA	1

**Table 12:** Association between haplotypes of ABCC2_rs8187710/SULT1A1_rs3760091/SULT1A1_rs4788068/SULT1C4_rs1402467 and the excretion of phase II flavanone metabolites in urine of studied volunteers after single dose of orange juice.

Haplotype Association with Response							
	rs8187710	rs3760091	rs4788068	rs1402467	Freq	OR (95% CI)	*p*-Value
1	G	C	C	C	0.3905	1	---
2	G	G	C	C	0.2092	**0.07 (0.01–0.81)**	**0.039**
3	G	C	T	G	0.1648	**0.01 (0.00–0.17)**	**0.0038**
4	G	G	T	G	0.0794	0.52 (0.04–6.58)	0.61

In bold the significant difference *p* < 0.05.

## Data Availability

The data from this study will be available up request.

## Supplementary Materials

The following supporting information can be downloaded at: https://www.mdpi.com/article/10.3390/nu14183770/s1, Table S1: Exact test for Hardy-Weinberg equilibrium (*n* = 46).
