# Supplementary material for: Association between Single Nucleotide Polymorphisms of SULT1A1, SULT1C4, ABCC2 and Phase II Flavanone Metabolites Excretion after Orange Juice Intake
Source: Nutrients. 2022 Sep 13;14(18):3770. doi: 10.3390/nu14183770 (PMC9502135; doi:10.3390/nu14183770)
Supplement: Supplementary file 1 [file nutrients-14-03770-s001.zip › nutrients-1911890-supplementary.pdf]

Supplementary Material

Table S1. Exact test for Hardy-Weinberg equilibrium (n = 46).

	N11	N12	N22	N1	N2	P-value
**rs8187710**						
	N11	N12	N22	N1	N2	P-value
All subjects	40	6	0	86	6	1
Status=0	16	5	0	37	5	1
Status=1	24	1	0	49	1	1
**rs3760091**						
	N11	N12	N22	N1	N2	P-value
All subjects	15	26	5	56	36	0,35
Metabolites=0	6	11	4	23	19	1
Metabolites=1	9	15	1	33	17	0,18
**rs4788068**						
	N11	N12	N22	N1	N2	P-value
All subjects	20	21	5	61	31	1
Metabolites=0	4	13	4	21	21	0,4
Metabolites=1	16	8	1	40	10	1
**rs1402467**						
	N11	N12	N22	N1	N2	P-value
All subjects	25	13	7	63	27	**0,039**
Metabolites=0	6	10	5	22	20	1
Metabolites=1	19	3	2	41	7	**0,047**

In bold the significant difference $p < 0.05$.
